# A Concept for Selection of Codon-Suppressor tRNAs Based on Read-Through Ribosome Display in an *In Vitro* Compartmentalized Cell-Free Translation System

**DOI:** 10.1155/2012/538129

**Published:** 2012-07-18

**Authors:** Atsushi Ogawa, Masayoshi Hayami, Shinsuke Sando, Yasuhiro Aoyama

**Affiliations:** ^1^Department of Synthetic Chemistry and Biological Chemistry, Graduate School of Engineering, Kyoto University, Katsura, Nishikyo-ku, Kyoto 615-8510, Japan; ^2^Ehime University, 3 Bunkyo-cho, Matsuyama, Ehime 790-8577, Japan; ^3^INAMORI Frontier Research Center, Kyushu University, 744 Motooka, Nishi-ku, Fukuoka 819-0395, Japan; ^4^Department of Molecular Chemistry and Biochemistry, Faculty of Science and Engineering, Doshisha University, 1-3 Tatara-Miyakodani, Kyotanabe, Kyoto 610-0321, Japan

## Abstract

Here is presented a concept for *in vitro* selection of suppressor tRNAs. It uses a pool of dsDNA templates in compartmentalized water-in-oil micelles. The template contains a transcription/translation trigger, an amber stop codon, and another transcription trigger for the anticodon- or anticodon loop-randomized gene for tRNA^Ser^. Upon transcription are generated two types of RNAs, a tRNA and a translatable mRNA (mRNA-tRNA). When the tRNA suppresses the stop codon (UAG) of the mRNA, the full-length protein obtained upon translation remains attached to the mRNA (read-through ribosome display) that contains the sequence of the tRNA. In this way, the active suppressor tRNAs can be selected (amplified) and their sequences read out. The enriched anticodon (CUA) was complementary to the UAG stop codon and the enriched anticodon-loop was the same as that in the natural tRNA^Ser^.

## 1. Introduction

Selection/amplification is a general tool for directed evolution of nucleic acids and proteins [1], which is much more complicated for reaction promoters (biocatalysts) than for simple binders [2]. Selection and identification for the former require some sort of catalyst-product pairing in an isolated compartment. *In vivo* selection using living cells has been typical choice for such a purpose. Meanwhile, *in vitro* selection, as opposed to *in vivo* selection, is simple and convenient to carry out, is free from cytotoxicity problems, and allows for starting with a library of great diversity. 

Griffiths and Tawfik proposed an *in vitro* compartmentalization (IVC) technique for the *in vitro* evolution of biomolecules including biocatalysts, where biocatalysts are transcribed/translated in a compartmentalized water-in-oil emulsion to allow catalyst-product pairing [2]. In practice, the IVC technique has been applied successfully for evolving or improving biocatalysts such as ribozymes and enzymes [3–12]. 

Here, we applied the IVC technique for evolution of tRNAs (one of catalysts in protein translation systems) [13–16] and present a promising concept for *in vitro* selection of suppressor tRNAs by the combination of read-through ribosome display (Rt-RD, *vide infra*) [17, 18].

## 2. Materials and Methods

### 2.1. General Design, Transcription/Translation System, and Analysis

Biological reagents and solvents were purchased from standard suppliers and used without further purification. Binding of suppressor tRNA to an amber stop codon is in competition with that of release factor 1 (RF1) to terminate the translation. The amber codon is also often misread by the exogenous tRNA for Gln. To maximize the suppression efficiency and minimize incorporation of Gln at the amber codon, we used a reconstituted prokaryotic cell-free translation system (PURESYSTEM Classic) [19], in which RF1, and Gln and Gln-tRNA synthetase had not been added unless otherwise stated. The T7-promoted translation is generally more efficient for longer templates. To keep a “balance” in the amounts of the fused mRNA (mRNA-tRNA) (longer) and the tRNA (shorter) transcribed, we put GCC immediately downstream of the first T7-promoter so as to lower the translation efficiency for the fused mRNA (It is known that a G-less sequence, immediately downstream of the T7 promoter, suppresses the transcription efficiency). Gel electrophoresis and blotting were carried out on a BE-250 electrophoresis apparatus (BIO CRAFT) and a Trans-Blot SD system (Bio-Rad), respectively.

### 2.2. Preparation of Template DNAs for Selection

pDHFR, encoding *E. coli* DHFR, was a gift from Dr. Y. Shimizu. The first PCR was carried out in 20 *μ*L of a reaction mixture containing 4 pmol of a forward primer containing a FLAG domain (bold) and a TAG amber stop codon (italic) 5′-d(AA GGA GAT ATA CCA ATG **GAC TAC AAG GAT GAC GAT GAC AAG** *TAG* ATC AGT CTG ATT GCG GCG TTA G)-3′, 4 pmol of a reverse primer with the lower T7 promoter (underlined) 5′-d(GTT CAG CCG CTC CGG CAT CTC TCC TAT  AGT  GAG  TCG  TAT  TAC CGG GTG ACT GCT GAG GA)-3′ for the tRNA-fused template or 5′-d(TGG CGG AGA GAG GGG GAT TTG AAC CGG GTG ACT GCT GAG GA)-3′ for nonfused template, 20 ng of pDHFR, 1.25 U of *Pfu Ultra* HF DNA polymerase (Stratagene), 4 nmol each of dNTPs (TOYOBO), and 2 *μ*L of 10 ×*Pfu Ultra* HF reaction buffer. After the PCR reaction, the product was purified by agarose gel electrophoresis. The second PCR was carried out in 20 *μ*L of a reaction mixture containing 4 pmol of a forward primer with the upper T7 promoter (underlined) and the following sequence after modification (vide supra) of the widely-used one 5′-d(T  AAT  ACG  ACT  CAC  TAT  AGC CCG GCC ACA ACG GCT GGG CTC TAG AAA TAA TTT TGT TTA ACT TTA AGA AGG AGA TAT ACC A)-3′, 4 pmol of a reverse primer with the sequence for *E. coli* tRNA for Ser (SerU) 5′-d(TGG CGG AGA GAG GGG GAT TTG AAC CCC CGG TAG AGT TGC CCC TAC TCC GGT L_1_L_2_X YZL_3_ L_4_AC CGG TCC GTT CAG CCG CTC CGG CAT C)-3′ (L_1~4_ and XYZ represent the anticodon-loop and anticodon bases, respectively), ~20 fmol of the purified first PCR product, 1.25 U of *Pfu Ultra* HF DNA polymerase, 4 nmol each of dNTPs, and 2 *μ*L of 10 ×*Pfu Ultra* HF reaction buffer.

### 2.3. *In Vitro* Selection of Suppressor tRNAs

*In vitro* coupled transcription/translation of template DNAs in the reverse-phase micelle was carried out as follows. A 50 *μ*L portion of a cell-free translation system containing 50 pM of a DNA template (2.5 fmol) was added gradually to 950 *μ*L of mineral oil (Sigma) containing detergents Span 85 (Nacalai Tesque) (4.5% v/v) and Tween 20 (Sigma) (0.6% v/v) under stirring on ice. The diameter (*d*) of the micelles (water droplets) in the resulting emulsion was about 2 *μ*m, indicating that the number of micelles was *N* = *V*/*v* = 1.2 × 10^10^, where *V* is the total volume of the water phase (50 *μ*L) and *v* is the volume of a micelle with *d* = 2 *μ*m. The dsDNA used (2.5 fmol) contains 1.5 × 10^9^ template molecules. This number is one-order of magnitude smaller than that of the micelles. Thus, each micelle is expected to encapsulate maximally one template.

**Figure 1:**
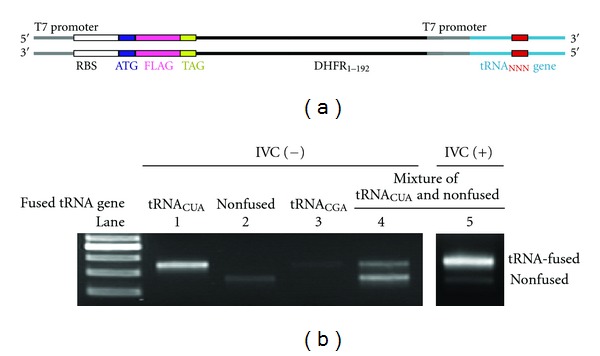
(a) Schematic sequence of the tRNA-fused template with two T7 promoters. The triple N in red represents the anticodon- or anticodon-loop-randomized region. (b) Agarose gel electrophoretic assay of the template DNAs recovered from tRNA_CUA_-fused template (lane 1), nonfused template lacking the tRNA domain (lane 2), a 1 : 1 mixture thereof (lanes 4, 5), or tRNA_CGA_-fused template (lane 3), after coupled transcription/translation under normal (noncompartmentalized) (lanes 1–4) or compartmentalized (lane 5) conditions.

After incubation of the mixture at 37°C for 1 h, the emulsion was spun at 2000 ×g for 10 sec. A 200 *μ*L portion of the supernatant was carefully taken from the upper part and mixed with 200 *μ*L of an ice-cold selection buffer (Phosphate-K, pH 7.3) containing 92.2 mM of K^+^, 300 mM of Na^+^, 50 mM of Mg^2+^, 0.05% of Tween 20 (WAKO), 2% of Block Ace (Dainippon Pharmaceutical Co.), and 1 mL of ice-cold water-saturated ether. The mixture was inverted twenty times and centrifuged at 16100 ×g for 10 min at 4°C, and then the organic ether phase was removed. The water phase was washed with 1 mL of ether and mixed with 200 *μ*L of ice-cold selection buffer. The mixture was applied on a column packed with prewashed anti-FLAG M2 agarose (Sigma) and gently inverted for 2 h at 4°C. After washing the gel retaining the ribosome-protein-mRNA (PRM) complex with 200 *μ*L of selection buffer five times, the mRNAs were eluted upon collapse of the RPM complexes with 200 *μ*L of an elution buffer (Phosphate-K, pH 7.2) containing 92.2 mM of K^+^, 300 mM of Na^+^, 30 mM of EDTA, 0.05% of Tween 20, and 2% of Block Ace. The mRNAs eluted were purified with an RNeasy MinElute Cleanup Kit (QIAGEN) and amplified with a QIAGEN One-Step RT-PCR Kit according to the manufacture's protocol using a forward primer with T7 promoter (underlined) and a FLAG domain (bold) followed by a TAG amber stop codon (italic) 5′-d(T  AAT  ACG  ACT  CAC  TAT  AGC CCG GCC ACA ACG GCT GGG CTC TAG AAA TAA TTT TGT TTA ACT TTA AGA AGG AGA TAT ACC A ATG **GAC TAC AAG GAT GAC GAT GAC AAG** *TAG*)-3′ and a reverse primer 5′-d(TGG CGG AGA GAG GGG GAT TTG AAC CCC CGG TAG AGT TGC C)-3′. The resulting RT-PCR products were used for the next round of selection and, after three (for anticodon-randomized tRNA) or five (for anticodon-loop-randomized tRNA) rounds, were monocloned using a PCR Cloning Kit (QIAGEN) and sequenced. Single-round coupled transcription/translation under normal (noncompartmentalized) or compartmentalized conditions (referring to Figure 1(b), lanes 1–4 or 5, resp.) was carried out using 400 pM of a template in 25 *μ*L of a cell-free translation system under otherwise identical conditions.

### 2.4. Preparation of Nonfused Template mRNAs for Western Blotting

The first PCR was carried out in 20 *μ*L of a reaction mixture containing 4 pmol of a forward primer with a FLAG domain (bold) with or without a TAG amber stop codon (italic) 5′-d(AA GGA GAT ATA CCA ATG **GAC TAC AAG GAT GAC GAT GAC AAG** [*TAG*] ATC AGT CTG ATT GCG GCG TTA G)-3′, 4 pmol of a reverse primer with an ochre stop codon (italic) 5′-d(TAT TCA *TTA* CCG CCG CTC CAG AAT CT)-3', 20 ng of pDHFR, 1.25 U of *Pfu Ultra* HF DNA polymerase (Stratagene), 4 nmol each of dNTPs (TOYOBO), and 2 *μ*L of 10 ×*Pfu Ultra* HF reaction buffer. After the PCR reaction, the product was purified by agarose gel electrophoresis. The second PCR was carried out in 20 *μ*L of a reaction mixture containing 4 pmol of a forward primer with T7 promoter (underlined) 5′-d(GAA ATT  AAT  ACG  ACT  CAC  TAT  AGG GAG ACC ACA ACG GTT TCC CTC TAG AAA TAA TTT TGT TTA ACT TTA AGA AGG AGA TAT ACC A)-3′, 4 pmol of a reverse primer 5′-d(TAT TCA TTA CCG CCG CTC CAG AAT CT)-3′, ~20 fmol of the purified first PCR product, 1.25 U of *Pfu Ultra* HF DNA polymerase, 4 nmol each of dNTPs, and 2 *μ*L of 10 ×*Pfu Ultra* HF reaction buffer. Template mRNAs were obtained by run-off transcription of the 5′-FLAG-TAG-DHFR or 5′-FLAG-DHFR template DNA obtained using a T7-MEGAshortscript Kit (Ambion). Thus, a T7-transcription mixture (10 *μ*L) containing 3 *μ*L of the PCR solution of template DNA was incubated at 37°C for 2 h. After addition of 1 U of DNase I, the mixture was incubated for additional 15 min. mRNAs were purified with an RNeasy MinElute Cleanup Kit (QIAGEN) and concentrations of the purified specimens were determined by the absorbance at 260 nm.

### 2.5. Preparation of tRNA^SerU^_L1L2XYZL3L4_

*E. coli *tRNA^SerU^_L1L2XYZL3L4_ was prepared by run-off *in vitro* transcription. Template DNA was prepared by PCR in 20 *μ*L of a reaction mixture containing 20 pmol of a forward primer with T7 promoter (underlined) 5′-d(G TAA  TAC  GAC  TCA  CTA  TA GGA GAG ATG CCG GAG CGG CTG AAC)-3′, 20 pmol of a reverse primer 5′-d(TGG CGG AGA GAG GGG GAT TTG AAC CCC CGG TAG AGT TGC C)-3′, 100 fmol of a template DNA (initial, RT-PCR amplified, or monocloned), 1.25 U of *Pfu Ultra* HF DNA polymerase (Stratagene), 10 nmol each of dNTPs (TOYOBO), and 2 *μ*L of 10 ×*Pfu Ultra* HF reaction buffer. The resulting PCR solution was used for transcription using a T7-MEGAshortscript Kit (Ambion) for 37°C for 20 h. The transcribed tRNAs were purified by denaturing PAGE (8%), followed by ethanol precipitation and, after dissolution in 500 *μ*L of water, further by passing successively through Microcon YM-30 (Millipore) and G-25 Microspin Columns (Amersham). Concentrations of the purified tRNAs were determined by the absorbance at 260 nm.

### 2.6. Translation of mRNA and Western Blotting Analysis with Exogenous tRNAs

Translation of a template mRNA (5′-FLAG-UAG-DHFR or 5′-FLAG-DHFR as a UAG(−) positive control) (2 *μ*g) was carried out at 37°C for 1 h in the presence of an exogenous tRNA (2 *μ*g) in 10 *μ*L of a reconstituted cell-free translation system, in which RF1 had not been added, but Gln and Gln-tRNA synthetase had. To the reaction mixture were added 165 *μ*L of water and 175 *μ*L of a sample-loading buffer (125 mM Tris-HCl (pH 6.8), 4% (w/v) SDS, 20% (w/v) glycerol, 0.002% (w/v) bromophenol blue, and 10% (v/v) 2-mercaptoethanol). The resulting solution was incubated at 95°C for 5 min and applied on 15% SDS-PAGE. Western blotting was performed on a PVDF membrane (Hybond-P, Amersham). FLAG-tagged proteins were visualized with anti-FLAG-HRP conjugate (Sigma) and ECL Plus Western Blotting Detection Reagent (Amersham). The suppression efficiencies were evaluated by comparing the band intensities of the FLAG-TAG-DHFR protein, determined by using the Image J software (NIH), with those of serially diluted (10, 20, 40, 60, 80, 100%) solutions of the stop-free FLAG-DHFR reference protein translated under otherwise identical conditions.

## 3. Results and Discussion

**Figure 2:**
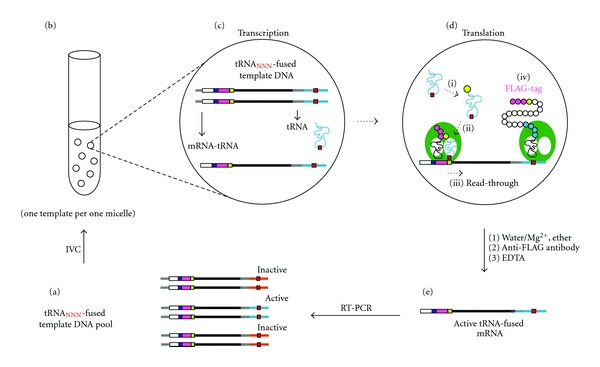
Rt-RD/IVC-based selection/amplification cycle. See the text for explanation.

We previously introduced Rt-RD [18], in which expressed protein could be fully displayed upon suppression of the stop codon(s) downstream of the open reading frame by appropriate suppressor tRNAs. In this paper, we conversely apply the Rt-RD technique for the selection of suppressor tRNAs that are coded at the 3′-terminus of the very template that contains the stop codon to be suppressed. We prepared a dsDNA template containing, under the control of a T7 promoter, the RBS (ribosome binding site), the ATG start codon, a FLAG domain, the TAG (amber) stop codon, and a spacer (DHFR_1–192_) derived from the *E. coli *DHFR gene covering the amino acids 1–64, followed by the tRNA sequence together with its own T7 promoter (Figure 1(a)). There are two T7 promoters in this template, transcription of which thus generates two types of RNAs; the one that contains both an RBS and an AUG serves as an mRNA, and the other, which lacks them, serves only as a tRNA. When the latter (tRNA) happens to suppress (read through) the amber (UAG) stop codon in the transcribed mRNA, a fused protein containing the FLAG, DHFR_1–192_, and tRNA regions would result upon translation and remain attached to the mRNA with the DHFR_1–192_-tRNA portion, serving as a spacer to be anchored in the ribosome tunnel to squeeze the FLAG-tag peptide for full display (Rt-RD; Figure 2).

We first confirmed that the right amber suppressor (tRNA^SerU^_CUA_) generated in this way worked as such. The anticodon-adjusted (CUA) tRNA^SerU^ is known to be aminoacylated with Ser by endogenous Ser-tRNA synthetase and hence suppress (read through) the amber codon with concomitant incorporation of Ser at that position [18, 20]. Coupled transcription/translation for 1 h of the template fused with the gene for tRNA^SerU^_CUA_ (Figure 1(a), NNN = CUA) in a reconstituted prokaryotic (*E. coli*) cell-free translation system [19] containing T7 RNA polymerase was followed by affinity selection (4°C and [Mg^2+^] = 50 mM) of the FLAG-tag peptide domain displayed (Rt-RD) in the protein-ribosome-mRNA (PRM) complex. The mRNA template coding the tRNA^SerU^_CUA_ sequence was recovered upon disruption of the ternary complex with EDTA, RT-PCR amplified, and identified as such (Figure 1(b), lane 1). However, the template was by no means recovered efficiently when it was lacking (nonfused) the tRNA domain (lane 2) or fused with anticodon-mismatched (CGA) natural tRNA for Ser (NNN = CGA) (lane 3). (In respect to the weak spots seen in lanes 2 and 3(Figure 1(b)), it is known that the stop codons can be suppressed to some extent (up to several %) in an RF1-minus translation system by misreading even in the absence of a suppressor tRNA.) In these cases, there is no generation of the correct tRNA for amber suppression and hence translation stops at the amber (UAG) codon, giving rise only to the FLAG peptide with no linkage to the genotype (mRNA). Conversely, when equal amounts of nonfused (suppression irrelevant) and tRNA^SerU^_CUA_-fused (relevant) templates were used, both templates were recovered (lane 4) (In respect to the apparently stronger spot for the nonfused template in lane4(Figure 1(b)), it is generally true that shorter templates are more easily amplified by PCR.) This is because suppressor tRNA^SerU^_CUA_ generated from the latter template can suppress the amber codon of the former. To avoid such a crossover, we needed to compartmentalize the reactions of each template using a water-in-oil emulsion system [3–12]. With this technique (see below, Figure 2), we could selectively recover the tRNA^SerU^_CUA_-fused template coding the active suppressor tRNA (lane 5) from the above mixture.

**Figure 3:**
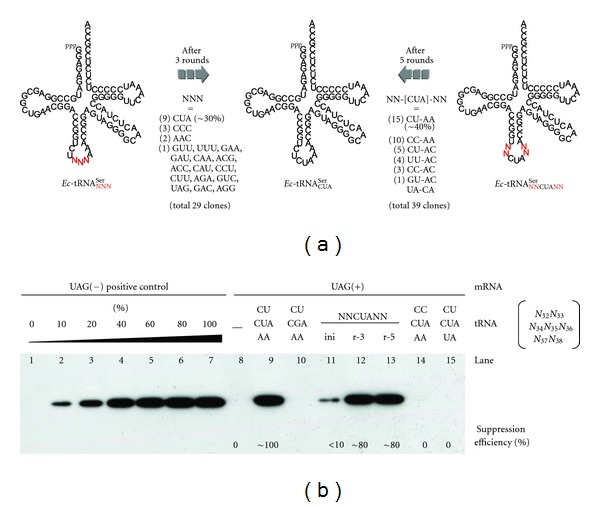
(a) Summary of selection/amplification of anticodon-randomized or anticodon-matched/anticodon-loop-randomized tRNAs for Ser. The numbers of clones that possess the sequence shown are indicated in parentheses. (b) Western blotting analysis of the translation of nonfused (tRNA-lacking) mRNA templates under normal (noncompartmentalized) conditions with (lanes 9–15) or without (lane 8) tRNA; tRNA^SerU^_CUA_ (lane9), tRNA^SerU^_CGA_ (natural tRNA for Ser, lane 10), anticodon-loop-randomized tRNA^SerU^ after 0, 3, or 5 rounds of selection/amplification (lanes 11, 12, or 13, respectively), or anticodon-matched but singly loop-mutated tRNA^SerU^ (lanes 14 and 15). Lane 7 represents a control translation using amber-free mRNA with no use of tRNA as a positive control and lanes 1–7 are a calibration set.

We then moved on to the selection of suppressor tRNAs. The selection cycle is shown in Figure 2. An initial pool (a) of fused templates (Figure 1(a), NNN = NNN with a diversity of 4^3^ = 64) with genes for anticodon-randomized tRNA^SerU^ (Figure 3(a), left) was subjected to coupled transcription/translation in a water-in-oil emulsion (b) [3–12]. Consequently, each compartment (~2 *μ*m) generates and contains a pair of template-related sister RNAs, a fused mRNA (shown as mRNA-tRNA) and a tRNA (c). The whole protein, susceptible to Rt-RD in the form of a PRM complex (step iv in d), can be translated (d) only when the tRNA serves as an amber suppressor (steps i–iii in d). The ternary complex thus formed was recovered by affinity selection of the displayed FLAG tag after breaking the emulsion and treated with EDTA to afford the active tRNA-fused template mRNAs (e), which were purified and amplified by RT-PCR back to the dsDNA templates (a), for use in another selection cycle. The DNAs obtained after three such cycles were monocloned and sequenced. The anticodon of approximately one third of the 29 monocloned tRNAs was CUA (Figure 3(a), center), which was the one expected based on codon-anticodon complementarity. These results indicate that the sensitive PRM complex survives the IVC and workup conditions to allow the Rt-RD/IVC method to select suppressor tRNAs.

Finally, we applied the Rt-RD/IVC method to the engineering of the two-base loop regions adjacent to the anticodon. These regions are variant in various tRNAs but are believed to be important in stabilizing codon-anticodon interactions [21]. Selection of fused templates with genes of anticodon-matched (CUA) and anticodon-loop-randomized (NN-CUA-NN with a diversity of 4^4^ = 256) tRNA^SerU^ (Figure 3(a), right) was carried out as above. DNA templates recovered after five cycles were monocloned and sequenced. Interestingly, ~40% of the 39 monocloned tRNAs had the same loop sequence (CU-CUA-AA) as the natural tRNA for Ser (CU-CGA-AA) even in the base modification-free conditions (Figure 3(a)). These results indicate that the anticodon-loop sequence itself has played a *preserved* or *sophisticated* role in the evolution of tRNAs that now have many modified bases (Since neither anticodon nor anticodon-loop region is recognized by the serenyl-tRNA synthetase [22], the selected loop sequence may play a role in stabilizing the interaction between the amber stop codon andthe suppressor anticodon.) [22, 23]. Suppression efficiencies were evaluated by western blotting analysis using the nonfused template lacking the tRNA domain in an RF1-minus translation system under noncompartmentalized conditions (Figure 3(b)). Enrichment of active tRNA became notably pronounced after three rounds (lanes 11–13) (The reason for the high suppression efficiency (~80%) despite the low percentage amounts of the active tRNA^SerU^_CUA_ (10% in round 3 and 38% in round 5) is that excess amounts of tRNA^SerU^_CUA_ were used in the experiments for western blotting (Figure 3(b))) and the suppression efficiency of the monocloned tRNA_CU-CUA-AA_ showed ~100% activity (lane9) compared with the suppression-free translation using a UAG(–) reference template (lane 7). Interestingly, a single mutation in the anticodon-loop domain led to a dramatic loss of activity (lanes 14 and 15). This is in accord with the above argument.

In summary, a concept for *in vitro* selection of suppressor tRNAs is presented. An essential aspect of it is that tRNA is a part of mRNA (mRNA-tRNA fusion), being located downstream of the particular codon to be suppressed (the amber stop codon in this study). Therefore, only active tRNAs that can *self-suppress* the codon in a water-in-oil compartment are susceptible to amplification by the read-through ribosome-display technique. 

Although the concept was well demonstrated, the present method must be further optimized especially regarding the selection efficiencies. Three or five rounds of selection were required for enrichment from the small library sizes (*n* = 64 and 256). One possible reason for this low selection efficiency is a misreading of stop codon in an RF1-minus translation system, which would result in the recovery of false positive tRNA-fused mRNAs. The misreading might be reduced by coexisting small amounts of RF-1. Another reason is the instability of RPM complex against extraction conditions. Optimization of extraction conditions or the use of mRNA display technique [24, 25] might overcome the instability. Of course, compartmentalization conditions or mRNA/tRNA ratios are also the factor to be checked.

Furthermore, it is not easy to understand that 10 clones out of 39 (26%) had the CC-CUA-AA anticodon-loop sequence after 5 rounds (Figure 3(a)), which turned out to be completely inactive in suppression (Figure 3(b), lane 14). Taking into account the fact that the CC-CUA-AA occupies 25% of pool even in round 3 in contrast to the less percentage amounts of the active CU-CUA-AA sequence (10%), it is feasible to speculate that the inactive CC-CUA-AA sequence might be already abundant in the initial pool and/or has been more easily amplified regardless of the selection process.

 After sufficient improvements, the method may provide a promising *in vitro* tool for tRNA evolutions.
